# Pore architecture and particulate organic matter in soils under monoculture switchgrass and restored prairie in contrasting topography

**DOI:** 10.1038/s41598-021-01533-7

**Published:** 2021-11-09

**Authors:** Archana Juyal, Andrey Guber, Maxwell Oerther, Michelle Quigley, Alexandra Kravchenko

**Affiliations:** 1 Department of Plant, Soil and Microbial Sciences, Michigan State University, East Lansing, MI 48824 USA; 2 Department of Horticulture, Michigan State University, East Lansing, MI 48824 USA

**Keywords:** Biogeochemistry, Ecology, Imaging techniques

## Abstract

Bioenergy cropping systems can substantially contribute to climate change mitigation. However, limited information is available on how they affect soil characteristics, including pores and particulate organic matter (POM), both essential components of the soil C cycle. The objective of this study was to determine effects of bioenergy systems and field topography on soil pore characteristics, POM, and POM decomposition under new plant growth. We collected intact soil cores from two systems: monoculture switchgrass (*Panicum virgatum* L.) and native prairie, at two contrasting topographical positions (depressions and slopes), planting half of the cores with switchgrass. Pore and POM characteristics were obtained using X-ray computed micro-tomography (μCT) (18.2 µm resolution) before and after new switchgrass growth. Diverse prairie vegetation led to higher soil C than switchgrass, with concomitantly higher volumes of 30–90 μm radius pores and greater solid-pore interface. Yet, that effect was present only in the coarse-textured soils on slopes and coincided with higher root biomass of prairie vegetation. Surprisingly, new switchgrass growth did not intensify decomposition of POM, but even somewhat decreased it in monoculture switchgrass as compared to non-planted controls. Our results suggest that topography can play a substantial role in regulating factors driving C sequestration in bioenergy systems.

## Introduction

Perennial bioenergy crops are a promising cellulosic feedstock because of their ability to thrive under low nutrient inputs and to grow on marginal lands which makes them ideal for providing long term accumulation of soil C and for reducing greenhouse gas emissions^1,2^. Perennial grasses have high potential for soil C sequestration as they have extensive root systems that can contribute large amounts of C belowground^3^. But there are some exceptions, i.e., switchgrass (*Panicum virgatum*, L.), a bioenergy crop well regarded for its durability and high biomass production^4^. Despite a large root system switchgrass tends to be slower in enabling soil C gains compared to native succession vegetation^2,5^. For example, five years post establishment perennials polycultures, poplar and native succession with high plant diversity had 2.5 times greater active C pools than perennial monocultures, i.e., switchgrass and miscanthus, and annual cropping systems, e.g., continuous corn, in a moderate fertile soils^2^. However, switchgrass can be a strong positive contributor to soil C gains when grown within a polyculture prairie community^6^.

The soil C stocks are maintained by a fine balance between several contributing factors, including plant shoot and root production, root exudation, and microbial decomposition^7^. Soil pore structure is yet another key factor that plays an important role in maintaining and increasing soil C. Pores regulate transport of nutrients and microbes, and control water and air flows^8,9^. Plant roots are important drivers of soil pore structure formation^10^ and root effects can vary depending on the root morphology. Bodner et al.^11^ reported a 30% increase in macroporosity (> 37.5 µm) due to presence of plants with coarse root systems and a greater volume of micropores (< 15 µm) in plant species with fine root systems. Bacq-Labreuil et al.^12^ demonstrated that different cover crop species had contrasting effects on soil porosity, pore connectivity, and microbial communities. Soils under monoculture switchgrass systems also differ from diverse plant communities in their pore characteristics, with the latter having noticeably greater presence of pores in 15–75 µm radius range^13^.

Particulate organic matter (POM) is a key component of soil C stocks. POM consists of plant and animal remnants at various stages of decomposition ranging in size from 0.054 to 2 mm^14^. POM particles can comprise anywhere from 6 to 37% of the total soil organic C (SOC) in agricultural soils^15^. POM persists in soil through physical protection when it is spatially inaccessible to decomposers and through its inherent biochemical recalcitrance^16,17^. Physical protection of POM from decomposers is enhanced by practices like no-till management and perennial vegetation. However, root exudates can stimulate POM decomposition through positive priming effect^18^. Studying the influence of new roots on POM and its potential decomposition in bioenergy cropping systems will provide a better understanding of bioenergy crops contributions to soil organic matter stabilization.

Besides plant diversity, topography greatly influences spatial patterns in soil C and nitrogen. Topographical factors, such as landscape position, slope gradient, and elevation, control redistribution of minerals and water affecting, in turn, soil microbial communities and SOC decomposition^19,20^. Most of the studies of soil C sequestration so far have focused either on the effects of bioenergy cropping systems or on the effects of topography, while analyses of the combined effects of bioenergy cropping and topography on soil pore characteristics and soil C are limited. Bioenergy crop production targets marginal lands to avoid competition for the available land with food production^1,4^. Areas with contrasting topography, e.g., steep eroded slopes or often flooded undrained depressions, are among marginal lands suitable for bioenergy cropping. Understanding C sequestration performance of different bioenergy systems and factors that influence it in topographically diverse terrain is needed for maximizing benefits from bioenergy cropping on marginal lands.

In this study, we address the impacts of bioenergy cropping systems at contrasting topographies on soil pore structure and stability of POM. The first objective of the study was to investigate soil pore architecture and decomposition of POM in soils from two bioenergy cropping systems (i.e., a monoculture switchgrass and a restored prairie), at multiple locations from two contrasting topographical positions, namely, depressions and relatively steep slopes. Non-invasive techniques, like X-ray computed micro-tomography (µCT), enable determination of key soil pore characteristics such as porosity, pore numbers, sizes, connectivity, and tortuosity^21–24^. Combing µCT with soil biological and decomposition experiments provides a better understanding of the role that pore architecture plays in biological and chemical process at micro-scale^25–27^. Our second objective was to explore which soils, from monoculture switchgrass or prairie, and from what topographical position provide better environment for POM protection when subjected to new plant growth. Repeated µCT scanning of intact soil samples prior and after new plant growth offers unique opportunities to quantify decomposition in POM fragments^28,29^.

We hypothesize that (1) after 10 years of implementation of the two cropping systems there will be a greater presence of pores in 30–90 µm size range and a concomitant increase in soil C in prairie as opposed to monoculture switchgrass, (2) greater differences between the two plant systems in pore architectures and in C gains will be observed in topographical depressions due to their higher clay and silt contents beneficial for soil C protection and pore formation, and (3) presence of live growing plants will stimulate decomposition of inherent soil POM due to positive priming induced by live plant roots and associated enhanced microbial activities.

## Materials and methods

### Study site, soil sampling, and experiment outline

Soil samples were collected from the scale-up experimental site at Marshall farm of the Great Lakes Bioenergy Research Center, Kellogg Biological Station, Michigan, USA (85°19″ W, 42°26″ N). The site was established in spring 2010. The soil is well-drained mixed, mesic Typic Hapludalf (Kalamazoo series) formed on glacial outwash. Permission to use the farm was obtained from the institution. Soil samples were collected in fall of 2018 from two long-term bioenergy cropping systems: monoculture switchgrass (*Panicum virgatum L*.) and restored prairie (an 18-species assemblage dominated by *Elymus canadensis*, *Schizachyrium scoparium*, *Sorghastrum nutans*, *Rudbeckia hirta*, and *R. triloba*) (Full list here: https://data.sustainability.glbrc.org/datatables/421).

**Figure 1:**
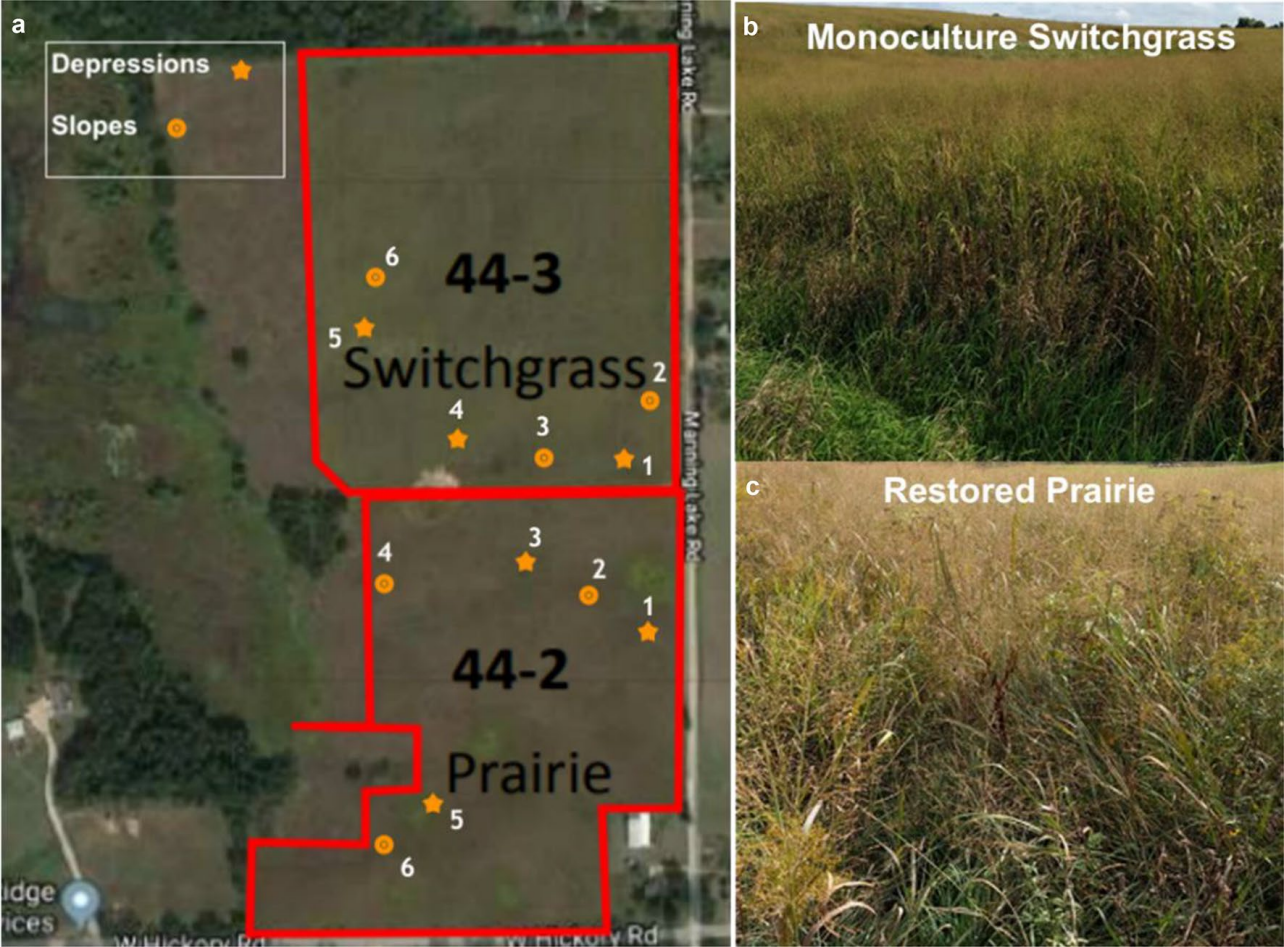
Experimental site and (**a**) sampling locations in two topographical positions from the two studied bioenergy cropping systems: (**b**) monoculture switchgrass and (**c**) restored prairie.

From each system, we collected soil samples from two contrasting topographical positions: topographical depressions representing footslopes and toeslopes, referred to further on as depressions, and uphill positions representing shoulders and backslopes, referred to as slopes. For each cropping system we sampled three stand-alone depression sites and three slope sites adjacent to the depressions (Fig. 1). At each sampling location we collected two intact soil cores (5 cm in diameter × 5 cm in height) from 5 to 10 cm depth along with the loose soil surrounding the intact cores. The intact cores were closed with foil caps from both ends, wrapped in aluminum foil, and stored together with loose soil at 4 °C. The collected loose soil was sieved through 8 mm and then 2 mm sieve to recover stones and roots. Roots recovered in the sieve were washed with distilled water and oven-dried for 3 days at 60 °C. The dry roots were then weighed using a precision scale. Total C and nitrogen (N) were measured in sieved and ground loose soil by combustion analysis on Costech Analytical Elemental Combustion System model 4010 for CHNS-O elemental analysis and Nitrogen / Protein determination (Costech Analytical Techologies, USA). Soil texture was determined by the hydrometer method^30^.

The outline of the experimental work is as following: first, the intact soil cores were subjected to X-ray µCT scanning to characterize soil pore architecture and POM and to explore the differences between topographical positions and bioenergy systems. Then, the cores were subjected to the 3-month switchgrass growth experiment, followed by the second X-ray µCT scanning. Comparing the µCT images before and after the switchgrass growth experiment enabled assessments of the role of new plant growth on POM decomposition and on pore architecture changes.

### Plant growth experiment

The experiment consisted of growing switchgrass in half the studied intact X-ray µCT scanned cores, referred to as planted treatment, while keeping the other half of the cores unplanted, referred to as no-plant control. One of the two cores from each sampling location was randomly selected for one of the two treatments. Two–three switchgrass (variety Cave-in-rock- plant material was identified by Maxwell Oerther) seeds were placed in each planted treatment core. After the seedlings germinated and established, only one plant per core was kept. Both planted and no-plant control cores were kept in the greenhouse and watered daily to maintain constant moisture content of 45–50% WFPS, with daily water losses determined by weighing. Average temperatures of 25 °C during the day and 22 °C at night were maintained in the greenhouse. After 3 months of growth, the plants were terminated, and the soil cores were wrapped in aluminum foil and stored at 4 °C until subsequent second scanning.

### X-ray µCT scanning

Soil cores were scanned using an X-ray μCT system (North Star Imaging, X3000, Rogers, USA) in the Department of Horticulture at Michigan State University. The cores were scanned at a resolution of 18.2 μm with energy settings of 75 kV and 450 μA with 2880 projections. The X-ray μCT images were reconstructed using efX software (North Star, Rogers, USA) and exported as Image stacks (*.TIFF format). Scanning of the soil cores was performed twice, first shortly after field collection, and then again after the described above plant growth experiment. Prior to the second scanning the soil cores were brought to the same moisture content (45–50% WFPS) level as during the first scanning. To achieve the same moisture content, soil cores were first allowed to gradually moisten by keeping them on a water-saturated coarse sand for 24 h. Then the cores were weighed and either subjected to air-drying or additional distilled water was added as required to achieve the prior-to-scanning moisture content.

### Image analysis

Image analyses for characterization of the soil pores and POM within the intact cores were performed in ImageJ (v1.5) software^31^. Prior to image analysis, the X-ray μCT images were preprocessed to remove random noise and scanning artifacts. Specifically, Remove Background tool of Xlib/Beat plugin^32^ was used to remove shadowing effects from the scanner. The tool was used to fit a third-degree global polynomial equation to the original image. The values obtained from the polynomial functions were then subtracted from the original image and the greyscale values were adjusted with the original image. After the background removal, a 3D Median filter with a two-voxel radius in all directions was applied to remove random noise. Contrast of the images was enhanced using Enhance contrast tool with 0.6% saturated pixel setting.

A region of interest of size 3.2 cm × 3.2 cm × 0.4 cm (1756 × 1756x221 pixels) was selected from the central portion of each μCT image to avoid ring artifacts close to the edges of the cores. Three subsamples of the given region of interest size were cropped from each soil cores. The processed images were segmented into solid and pore using adaptive window indicator kriging method^33^. Stone/gravel fragments > 2 mm were present in most of the μCT images. Most stones had markedly higher greyscale values as compared to the bulk soil which made it possible to segment them out using the thresholding method described above. After thresholding, the large stone fragments (> 10 mm^3^) were quantified using the Particle Analyzer tool in BoneJ^34^. The volume of the stones was excluded from further calculations of pore and POM volumes, that is, visible pores and POM characteristics are reported on a stone-free basis.

**Figure 2:**
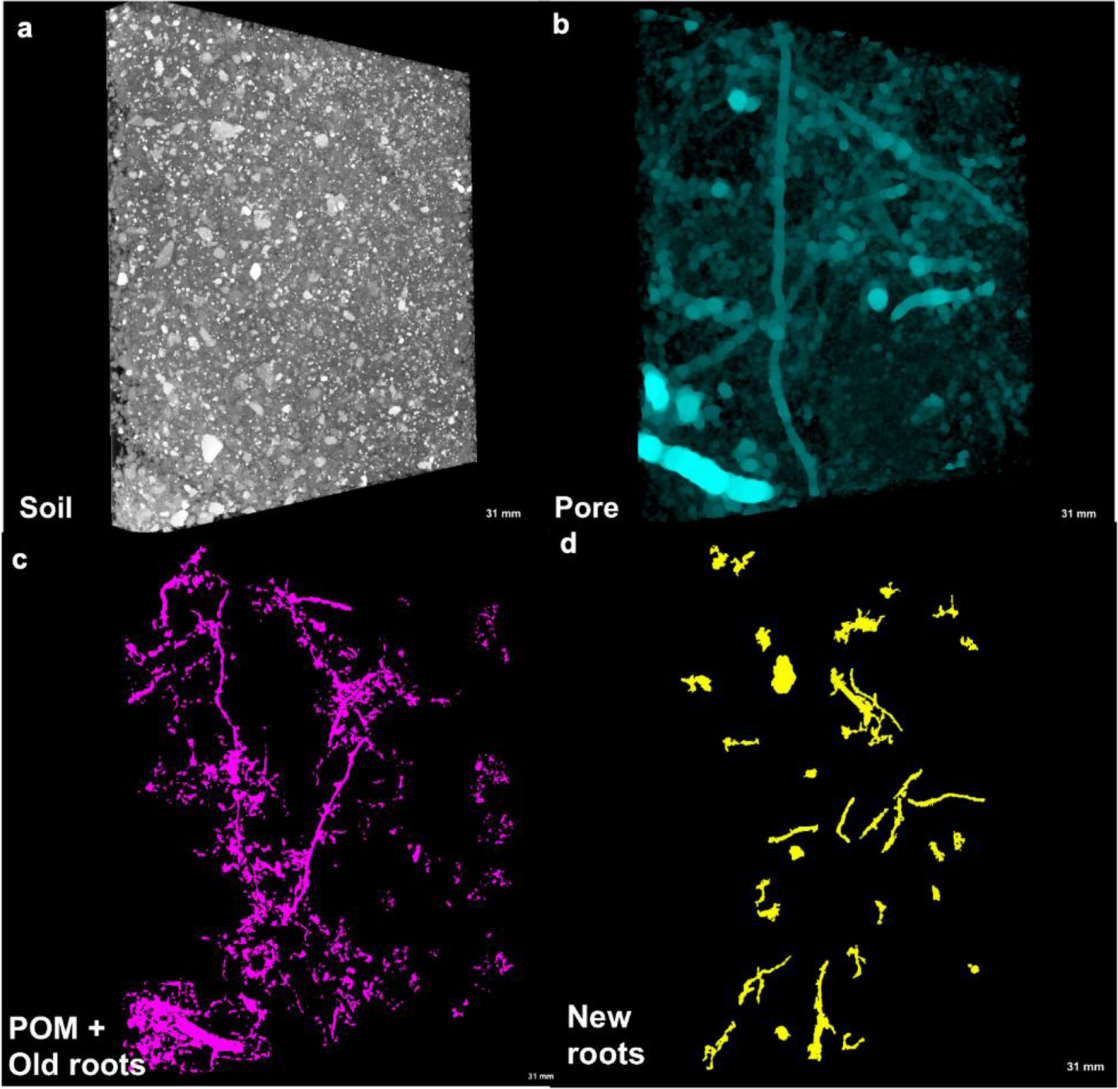
An example of μCT images from switchgrass cropping system μCT scanned at 18.2 μm resolution: (**a**) a 3D grayscale image, (**b**) segmented pores, (**c**) identified particulate organic matter, and (**d**) roots of a newly grown switchgrass plant. Image size is 3.2 × 3.2 × 0.4 cm.

Segmented images were used to determine soil pore characteristics, including visible porosity (> 18.2 µm), pore connectivity, and solid-pore interfacial area using the 3D Minkowski functionals as described in Houston et al.^35^ before and after the plant-growth experiment. Total porosity was calculated based on the bulk density of each sample, as determined from its weight and volume, with particle density of 2.6 g cm^−3^. Visible porosity was obtained as the fraction of the total stone-free image volume occupied by > 18.2 µm diameter pores. Volume of < 18.2 µm pores was calculated as the difference between the total and visible porosities. Solid-pore interfacial area was determined as an area of solids directly bordering the pore space. Pore connectivity was estimated as the fraction of the pore volume connected to the external surface of the image. Pore size distribution was determined using Xlib plugin for ImageJ^32^. In this tool, the continuous 3D pore-size distribution option was selected which fits spheres of maximum radii within pore space using the maximal inscribable sphere method (Fig. 2).

POM was analyzed using the method described in Kravchenko et al.^13^. Briefly, we manually selected representative POM fragments from each core and obtained their minimum and maximum grayscale values. Then averages of minimum and maximum greyscale values from the fragments were calculated for each core and then used as lower and upper thresholds to segment all POM fragments within the core. The images were then subjected to a series of 3D erode/dilate steps to remove the misclassified voxels which were typically located on the boundaries of the solids and pores. Particle analyzer tool of BoneJ plugin^34^ was used to select the POM pieces greater than 0.018 mm^3^, followed by 3D Gaussian filtering and segmentation.

To get a more in-depth view at the effect of the plant growth on POM decomposition, 3–5 POM fragments were selected within each core. The selection of POM fragments was based on their grayscale value, size and shape characteristics. Specifically, the POM fragments that had a better contrast from the soil background and clear sharp edges were used. We quantified the size of each fragment on before- and after-plant growth µCT images following the image analyses steps described above. The amount of POM decomposed after the plant growth experiment was calculated as

1 $$Amount~of~POM~loss=\left(1-\frac{{A}_{a}}{{A}_{b}}\right)\times 100$$

where *A*_*b*_ is the percent of POM voxels as determined on the initial µCT image, *A*_*a*_ is the percent of POM voxels on the image after the plant growth experiment.

For each of the selected POM fragments we determined the presence of pores within certain distances from the POM. A 3D dilation tool of BoneJ plugin was used to create a layer around each POM fragment to cover distances of 1 mm, 5 mm, and 8 mm. We overlaid µCT pore images with masks of POM distance layers to identify the pores in vicinity of POM fragments. The fraction of pores within each layer was calculated as the number of voxels occupied by the pores within the layer divided by the total number of voxels within that layer.

For POM fragments in the cores from the planted treatment we also quantified the presence of new switchgrass roots in the vicinity of each POM fragment. For that we overlaid binary images of roots with the POM images and measured the distances from the POM fragments to the roots using the Distance Transform tool in ImageJ.

### Statistical analysis

Comparisons between the studied bioenergy cropping system, topography, and plant growth treatments in terms of soil and pore characteristics and POM were conducted using the mixed model approach implemented in the PROC MIXED procedure of SAS version 9.4 (SAS, USA). The statistical model to assess the effects of cropping systems and topography on field soil characteristics, e.g., soil C and N, gravimetric soil moisture at field sampling, consisted of fixed effects of system and topography, and their interaction (Table S1). Since soil texture was expected to be a major influence on soil organic matter, for soil C and N we also conducted an analysis of covariance (ANCOVA) with sand as the covariate (Table S2) following the approach outlined in Milliken and Johnson^36^. The statistical model to analyze soil pore characteristics measured before and after plant growth experiment consisted of fixed effects of system, topography, planting treatment, and their interaction and a random effect of the soil cores nested within the system, topography, and planting treatments^36^. ANCOVA was used to examine associations between soil pore characteristics and POM decomposition^36^.

The assumptions of normality and variance homogeneity were assessed using normal probability plots and side-by-side box plots of the residuals, followed by Levene's test for unequal variances. In cases where normality assumption was not met, e.g., for image-based porosity and pore connectivity, the data were log-transformed. The interaction effects were examined using slicing, aka F-tests for simple effects, with further mean separations using t-tests conducted when slice F-tests results were statistically significant at 0.05 level. The differences among the studied treatments that were not statistically significant at 0.05 level, but were consistent with the study hypotheses, are reported further on as numeric differences.

### Ethical statement

Experimental research and field studies on plants (either cultivated or wild), including the collection of plant material, complies with relevant institutional, national, and international guidelines and legislation.

## Results

### Soil characteristics

**Figure 3:**
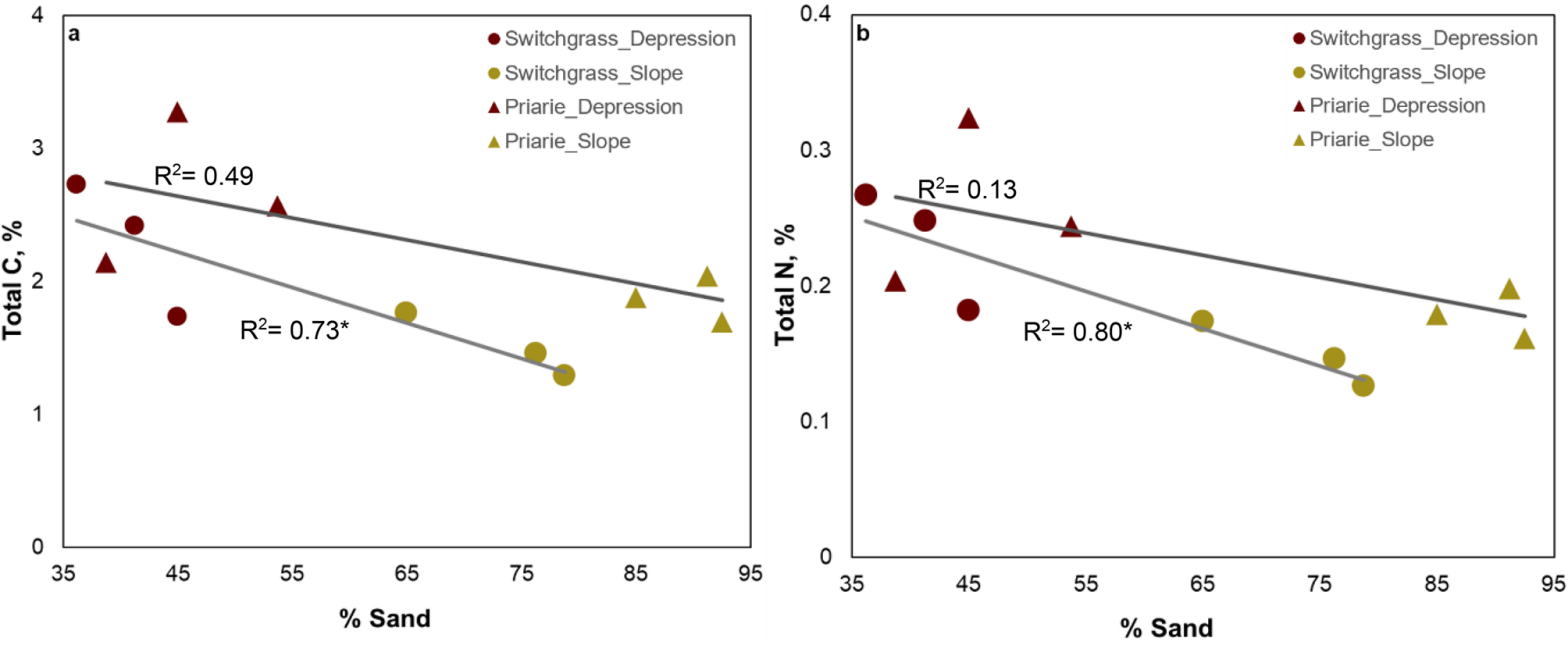
Total soil C (**a**) and N (**b**) plotted vs. sand content across depression (red) and slope (yellow) soils in prairie (▲) and switchgrass (●) systems. R2 value marked with * are significant at p < 0.05. Total C and N were significantly higher in prairie than in switchgrass (ANCOVA with sand as a covariate, p < 0.05 and p < 0.1 for C and N, respectively).

Soil texture differed predictably between the two topographical positions with depressions having lower sand content, but higher silt and clay contents than slopes (Table 1 and Table S1). In slopes soil texture differed between the two cropping systems—prairie had significantly higher sand and lower silt contents compared to switchgrass system. Total C and N concentrations were significantly higher in soils from depressions than in slopes and, based on ANOVA, only numerically higher in prairie than in switchgrass (Table. 1). In both topographical positions, C and N concentrations were negatively correlated with sand content (Fig. 3). Upon accounting for the variations in sand content via ANCOVA the total soil C and N under prairie vegetation were found to be significantly higher than those under monoculture switchgrass (Table S2).

**Table 1:** Summary of basic soil characteristics for the studied prairie and monoculture switchgrass systems at the two topographical positions.

Topography	System	Bulk-density (g cm^-3^)	Sand (%)	Silt (%)	Clay (%)	GWC (%)	Stones (g kg^-1^ of soil)	Roots (g kg^-1^ of soil)	Total C (%)	Total N (%)	POM (%)
Depression	Prairie	1.29	46a	46a	8a	22.3a	8.1a	6.7a	2.6a	0.2a	0.5
	Switchgrass	1.41	41a	53a	6a	20.4a	1.1a	3.5	2.2a	0.2a	0.6
Slope	Prairie	1.30	90*b	9*b	1b	11.2b	51.3b	30.5*b	1.8b	0.1b	0.6
	Switchgrass	1.45	73b	25b	2b	11.4b	64.3b	5.2	1.5b	0.1b	0.9
	Standard error	0.05	3.5	3.4	0.8	1.8	10.3	6.1	0.2	0.02	0.3

Shown are means (n = 6) and standard errors for each system and topographical position.

GWC is gravimetric water content at the time of sampling; POM is particulate organic matter.

Stars (*) indicate significant differences between cropping system within each topography (p < 0.05); letters indicate significant differences between the topographical positions within each cropping system (p < 0.05). No stars or letters are shown when the differences are not statistically significant.

Prairie had substantially higher root volumes in soil from slopes than those of either switchgrass or prairie in depression positions. No significant differences between the systems or topographical positions were observed for POM (Table. 1 and Table S1).

### Pore characteristics prior to plant growth experiment

**Figure 4:**
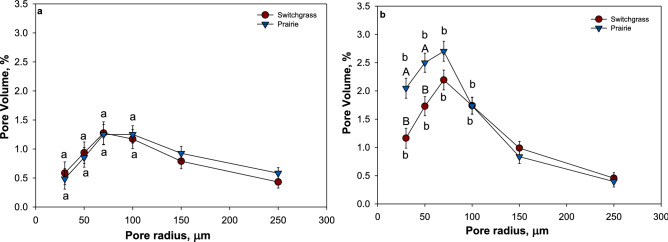
Pore size distribution for > 18.2 μm radius pores from topographical depressions (**a**) and slopes (**b**) in prairie (▼) and switchgrass (●) cropping systems before plant growth experiment. Shown are means with error bars representing standard errors (n = 6). Lower case letters indicate significant differences between topographical positions within each planting system (p < 0.05); upper case letters indicate significant differences between the cropping systems within each topographical position (p < 0.05).

In both plant systems topography significantly affected only the solid-pore interfacial area values, which were higher in soils from slope than in soils from depression (Table 2 and Table S3). Pore connectivity was significantly higher in soil from depressions than in slopes, but only in prairie system. Neither total nor image-based (pores > 18.2 μm) porosity was affected by topography in both plant systems. Pore size distributions differed between the two topographies and the abundance of pores in the 30–100 µm radius size range was significantly higher (p < 0.05) in soils from slope than in depression across both plant systems (Fig. 4).

**Table 2:** Summary of soil pore characteristics for the studied prairie and monoculture switchgrass systems at the two topographical positions before the plant growth experiment.

Topography	System	Total Porosity (%)	Pores < 18.2 um (%)	Pores > 18.2um (%)	Pore connectivity (%)	Solid-pore interface (mm^2^)
Depression	Prairie	50.5 (1.9)	32.9 (1.3)	17.5 (1.0)	94.6 (1.4)a	84.8 (8.5)a
	Switchgrass	46.2 (2.1)	31.7 (1.5)	14.5 (1.1)a	92.8 (1.5)	84.4 (9.3)a
Slope	Prairie	50.2 (1.9)	32.4 (1.3)	17.7 (1.0)	89.5 (1.4)b	184.5 (8.5)b*
	Switchgrass	44.6 (1.9)	27.1(1.3)	17.6 (1.0)b	92.7 (1.4)	134.3 (8.5)b

Shown are means (n = 6) and standard errors (in parentheses) for each system and topographical position.

Stars (*) indicate significant differences between the cropping systems within each topographical position (p < 0.05) and letters indicate significant differences between the topographical positions within each cropping system (p < 0.05). No stars or letters are shown when the differences were not statistically significant.

The plant system effect on pore characteristics was observed in soil from slopes, where solid-pore interfacial area was significantly higher (P < 0.01) in prairie system compared to switchgrass (Table 2). Total porosity was numerically higher in the prairie (50%) compared to the switchgrass (45%), both in depressions and slopes. Image-based porosities of prairie (17%) and switchgrass (16%) systems were very similar, while the percentage of small pores (< 18.2 μm) tended to be numerically lower in switchgrass than prairie in soil from both the topographies.

In soil from the slopes, the volumes of pores in 30–50 µm radius range were significantly higher in prairie compared to switchgrass (p < 0.05), while there was no significant difference in pore volumes between the two plant systems in depression (Fig. 4 and Table S4).

### Pore characteristics before and after plant growth experiment

After the plant growth experiment, all studied pore characteristics tended to decrease in control cores for every plant system and topographical position combination, however, the difference was not statistically significant (Table S5 and Fig. S1). In plant-grown cores, image-based porosity, and solid-pore interfacial area decreased in soils from every plant system and topography combination (Table S5). Pore connectivity on the other hand increased in soil from slopes for both plant systems, but the difference was not statistically significant. Change in volume of pores after the plant growth period was very minor in soils from every plant system and topography combination (Fig. S1).

### New plant growth and its effect on pore characteristics

**Figure 5:**
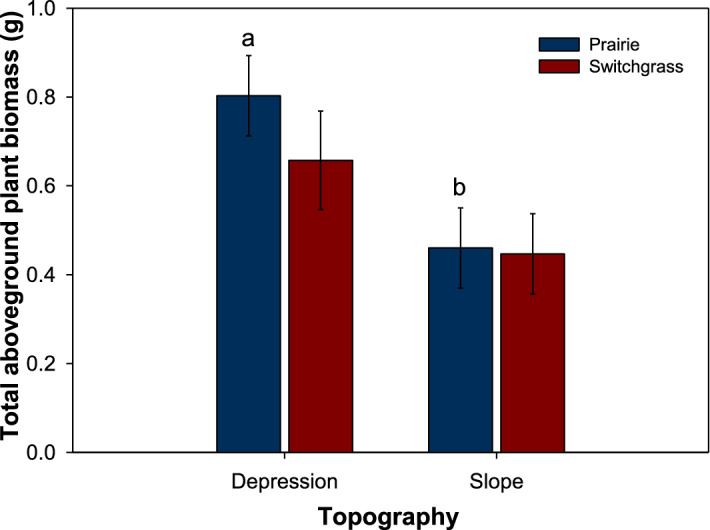
Total aboveground biomass of switchgrass plants grown in the studied soil cores from depression and slope topography in prairie (blue) and switchgrass (red) cropping systems. Data shown are means ± SE (n = 6). Letters indicate significant differences between topographical positions within each system.

The aboveground biomass of the newly grown switchgrass plants was numerically greater in prairie compared to monoculture switchgrass system (Fig. 5). The aboveground biomass was higher when the plants were grown in soil cores from depressions compared to slopes, however, this difference was statistically significant only in the prairie system.

**Figure 6:**
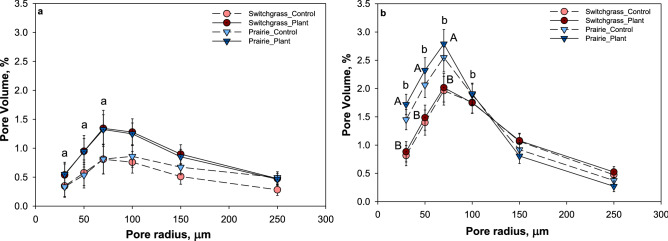
Pore size distribution for image-based (> 18.2 μm) pores for depression (**a**) and slope (**b**) in the control cores (solid lines) and in plant grown cores (dash lines) in prairie (▼) and switchgrass (●) cropping systems. Shown are means with error bars representing standard errors (n = 3). Lower case letters indicate significant differences between topographical positions within each system for plant-grown cores (p < 0.05); upper case letters indicate significant differences between cropping systems within each topographical position for plant-grown cores (p < 0.05).

In both plant systems, image-based porosity and pore connectivity were higher in plant-grown soil cores compared to control soil cores from depression. However, the difference was statistically significant only in soil cores from monoculture switchgrass system (Table 3 and Table S6). The volume of pores in 30–100 µm size range was also numerically higher in plant-grown cores compared to control cores (Fig. 6). In soil cores from slope, the difference between plant-grown and control soil cores in all studied pore characteristics was not statistically significant, and even the numerical difference was very minor for both plant systems.

**Table 3:** Summary of soil pore characteristics for the studied prairie and monoculture switchgrass systems at the two topographical positions after plant growth experiment.

Topography	System	Pores > 18.2 um (%)		Pore connectivity (%)	
	Treatment	Control	Plant	Control	Plant
Depression	Prairie	13.7	16.9	88.0	94.0
	Switchgrass	10.5*	16.0	78.6*	94.6
Slope	Prairie	16.9	16.8	91.4	92.0
	Switchgrass	16.5	17.9	92.8	93.2
	Standard error	0.01		0.02	

Shown are means (n = 6) and standard errors for each system and topographical position in control and plant-grown cores treatment.

Stars (*) indicate significant differences between plant-grown and control cores within each topography (p < 0.05) in each cropping system; upper case letters indicate significant differences between topographies in plant-grown cores (p < 0.05) in each cropping system and, lower case letters indicate significant differences between the cropping systems within each topographical position in plant-grown cores (p < 0.05). No * or letters are shown when the differences were not statistically significant.

**Figure 7:**
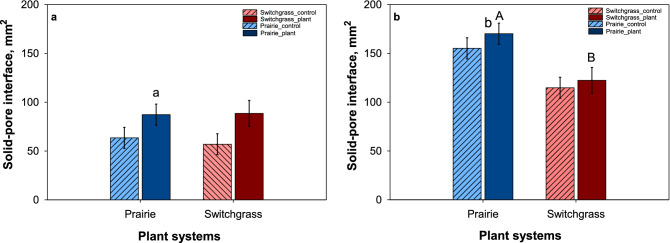
Solid-pore interface (mm^2^) for depression (**a**) and slope (**b**) in the control cores (dash) and in plant grown cores (solid) in prairie (blue) and switchgrass (red) cropping systems. Shown are means with error bars representing standard errors (n = 3). Lower case letters indicate significant differences between topographical positions within each system for plant-grown cores (p < 0.05); upper case letters indicate significant differences between cropping systems within each topographical position for plant-grown cores (p < 0.05).

In plant-grown soil cores from slope, the volume of pores in 30–70 µm radius size was significantly higher in prairie system compared to switchgrass. Between depressions and slopes, the abundance of pores in 30–70 µm radius size range was higher in soil cores from slope, however, the difference was statistically significant only in prairie system (Fig. 6). The plant system effect on solid-pore interfacial area was observed only in soil cores from slope where solid-pore interfacial area was significantly higher in prairie system compared to switchgrass. Within each cropping system, solid-pore interfacial area was higher in soil from slopes compared to depressions, however the difference was statistically significant only in prairie system (Fig. 7).

### Effect of new roots and soil pores on POM decomposition

**Figure 8:**
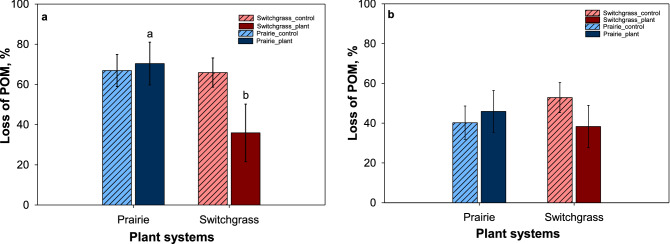
Amount of particulate organic matter (POM) decomposed in depression (**a**) and slope (**b**) topography in control cores (dash) and plant grown cores (solid) of prairie and switchgrass cropping systems. Data shown are means ± SE (n = 3). Letters indicate significant differences between cropping systems positions in control and plant grown cores in each topographical position (p < 0.05).

The amounts of POM decomposed after plant growth experiment varied between the two studied systems and topographical positions (Fig. 8). At both topographical positions, in switchgrass POM losses from control cores were numerically higher compared to that from plant-grown cores, while in prairie POM losses from control and plant-grown cores were almost identical. In plant-grown cores from depressions, the amount of POM decomposed was significantly higher in prairie system (p < 0.05) compared to switchgrass system (Fig. 8a). Similar pattern was observed in cores from slopes, but the difference was not statistically significant.

The amount of POM decomposed appeared to be positively associated with the size of the POM. That is, larger POM pieces tended to decompose faster in soil from both plant systems, but this correlation was not statistically significant (Fig. S2). No association between the presence of new roots and the amount of POM decomposed was observed in either prairie or switchgrass systems.

The presence of pores of certain sizes around POM that influenced the decomposition process differed in prairie compared to switchgrass system (Fig. S4). Within each system, the pattern in presence of pores of certain sizes that influenced POM decomposition was similar at all measured distances from the POM. In both plant systems from depressions, POM losses tended to increase with greater presence of 87–150 µm pores (Fig. S4c), but the correlations were not statistically significant. In prairie system from slopes, POM losses significantly increased with greater presence of 15–58 µm pores (Fig. S4b) and decreased with greater presence of 87–150 µm pores (Fig. S4d). An opposite trend was observed in soils from switchgrass system; however, the correlation was not statistically significant.

## Discussion

The experimental sites studied here are ten-year-old Conservation Reserve program (CRP) grassland fields converted to perennial cellulosic bioenergy plant systems. As expected, topographical position influenced the amount of C present in the soil with more C in low laying depressions than in upslope topography. Local variations in texture explained a substantial portion of variability in soil C and N with the amount of C expectedly negatively correlated with soil sand content (Fig. 3)^37,38^. Once the variability in soil texture was accounted for, the positive effect of diverse prairie vegetation on soil C and N became clearly visible (Fig. 3), supporting our hypothesis. This finding is consistent with a number of previous studies conducted both in vicinity of the current experimental site as well as in other climatic and edaphic settings^6,39,40^. However, the results did not support the hypothesis that greater positive effect of prairie will be observed in depressions with their fine-textured soils as compared to coarse textured slopes. In fact, an opposite took place, with differences between the two systems being the greatest when compared at high sand contents while minimal at low sand contents (Fig. 3). This is likely due to the interactive effect of topography on cropping system contributions, with prairie root production being 4.8 times higher than that in switchgrass monoculture in soils from slopes, but with two systems having similar root biomasses in depressions (Table 1). Higher root biomass productivity can be positively correlated with the soil sand content^41^. Greater positive effect of live vegetation on soil C in slopes than in depressions has been reported in the studied soils before. Ladoni et al.^37^ observed that after multiple years of including cover crops in the rotation, greater gains in labile soil C occurred in coarser-textured soils of eroded slopes and summits as compared to depressions.

The results of the pore-size distribution analyses showed that 10 years of prairie as a bioenergy cropping systems did lead to a greater presence of pores in 30–90 μm size range as compared to that in monoculture switchgrass but only in the soil from slopes (Fig. 4). The architecture of the pore system has changed as well, as attested by higher surface of the solid-pore interfacial area in prairie soil from slopes (Table 2). These two components, i.e. greater presence of 30–90 μm pores and larger surface area of solid-pore interface, appear to be crucial for promoting soil C gains. The pores of this size range create an optimal environment for microorganisms^42^, while large solid-pore interface provides opportunities for the microbial decomposition products and microbial necromass to encounter soil minerals and to become protected by physico-chemical bonds^43^. Enhanced C gains, which we observed in prairie soil on slopes (Fig. 3) concomitantly with improvements in soil pore characteristics (Fig. 4 and Table 2), are consistent with the concept of greater microbial spatial footprint on the surrounding soil as a promoter of soil C stabilization^5^.

High diversity of plant species in restored prairie is the likely reason for the observed enhanced pore development^5,13^. However, contrary to our expectations, the difference in presence of such pores between the two studied systems was greater in soil from slopes than in depressions. This result likely stems from the discussed above greater root biomass and greater C accumulation in prairie than in switchgrass soils. Roots are the primary driver of soil pore formation^11,44^, thus, their greater development on topographical slopes under prairie had an expectedly stronger effect on soil pore systems. However, soil C and pores are also related in a feedback loop; and greater organic matter accumulation means more intensive pore formation^45^.

The influence of live roots on pore development was demonstrated during our plant growth experiment through (i) a tendency for a greater volume of pores in 30–90 μm size range in plant-grown cores as compared to unplanted control cores (Fig. 6), and (ii) a tendency for greater solid-pore interface in plant-grown cores (Fig. 7). It appears that in control soils, the values of image-based porosity, pore connectivity, and solid-pore interface decreased during the time when the cores were kept watered along with the plant-grown cores (Table S5). The treatment of the control soil cores during plant-growth experiment can be regarded as a special case of laboratory incubation or a field fallow with optimized environmental variables, where intact cores were kept at varying but daily replenished to optimum soil moisture level and varying within optimal soil temperature range. Apparently, such regime when applied for > 2 months to intact soil from long-term perennial vegetation resulted in reduction of porosity, pore connectivity, and solid-pore interface. However, live switchgrass maintained these pore characteristics at the same levels as those from the original conditions. The difference in volume of pores in 30–90 µm range was greater in depression between plant-grown and control cores than in slopes. This is likely due to formation of more pores by roots of new plant in the cores from depressions whereas in slope cores, since those size pores were already present, roots of new plant would have grown into the existing pores.

POM decomposition was more affected by topography than by the plant system, across both plant-growth and control cores. Greater decomposition of POM in soil depressions is related to its higher C (Fig. 3) and, likely, greater microbial activity. Higher enzyme activity and microbial biomass in soils from topographical depressions than slopes of the studied area have been reported before^46^. The particularly high POM decomposition in prairie depressions was likely due to high microbial, both bacterial and fungal, activity in restored prairie as compared to monoculture switchgrass.

Presence of living roots is known to stimulate decomposition of previously stabilized soil organic matter by triggering microbial activity and causing priming^18,47^. Thus, we expected to see greater POM decomposition in the cores subjected to new plant growth. However, our hypothesis was not supported by the data. In the prairie soil, the presence of new roots did not significantly stimulate decomposition of inherent POM as compared to the control cores. On the contrary, in the cores from the switchgrass there was a noticeable numeric trend for lower POM decomposition in the cores with the plants as opposed to the control cores. The amount of POM decomposed in plant-grown cores from prairie system was 1.5 times greater than in switchgrass system, with the difference between the two systems being particularly pronounced in depression soils. It is possible that more diverse and active microbial communities of prairie soil^48^ were stimulated by new root growth, resulting in greater decomposition of inherent POM. Yet, it seems almost as in the soil from under long-term switchgrass monoculture the new switchgrass plants inhibited decomposition—the phenomenon that warrants further exploration. It should also be noted that the POM fragments randomly selected for this analysis from prairie soil cores tended to be located closer to the new root growth than in switchgrass soil (Fig. S5). Proximity to the new roots tended to be negatively associated with POM decomposition, where higher distances corresponded to lower decomposition (Fig. S5).

**Figure 9:**
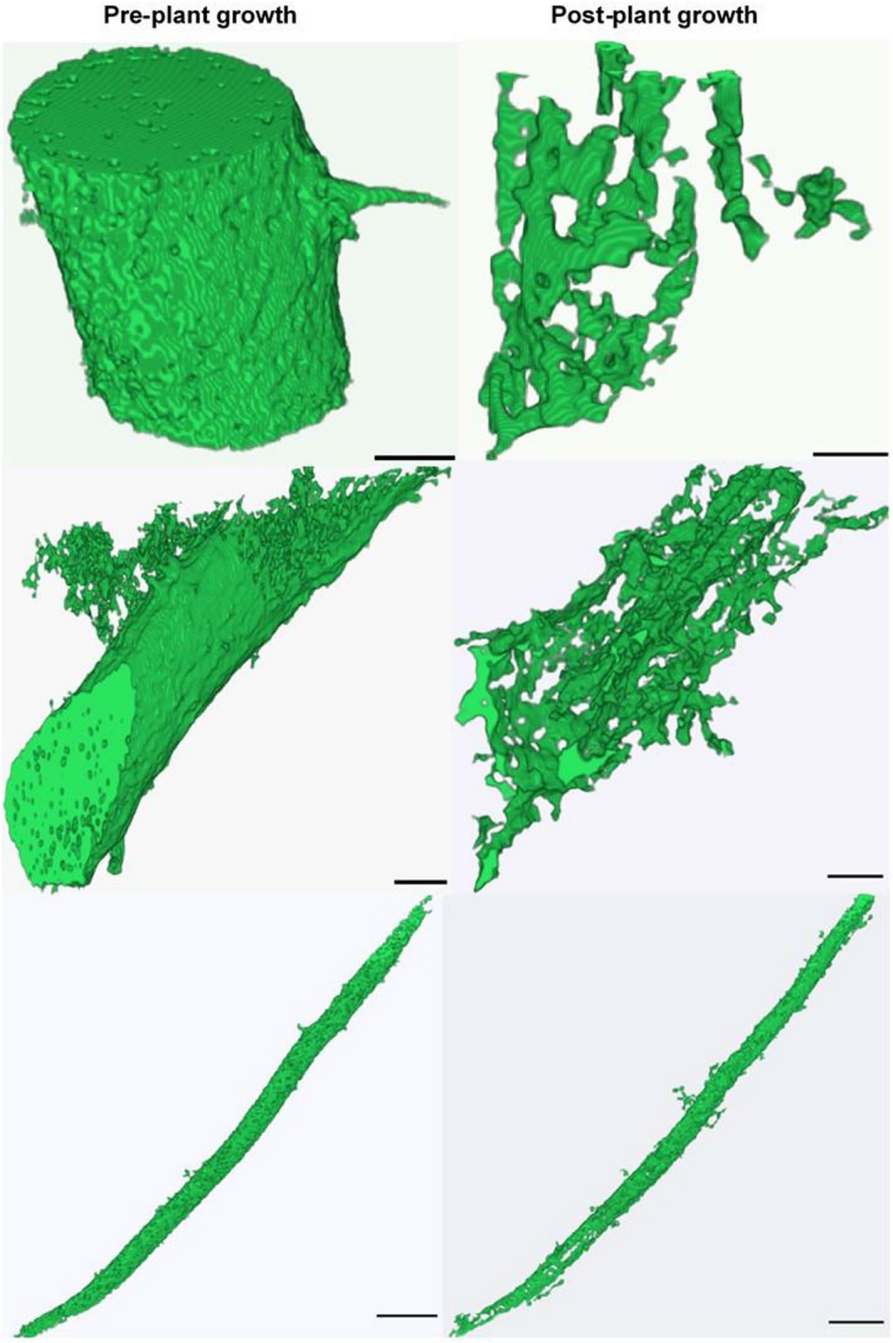
POM decomposition during the incubation period of plant growth experiment. Shown are examples of different types of POM fragments before (left) and after (right) plant growth incubation period.

The presence of pores of certain sizes that are associated with transport of new C inputs and access of microbial communities to such C rich sources could be another factor contributing to this decomposition of POM^26,49^. Our results showed that greater presence of pores in 15–58 μm size range caused increased decomposition of POM in prairie system as microbial decomposers gained access to the labile POM pieces. The percent of small size pores was higher in prairie system compared to monoculture switchgrass this explains why we observed a lower decomposition of POM in switchgrass. The size of POM pieces selection could also be a reason for this difference between prairie and switchgrass system as we observed bigger pieces to decompose faster (Fig. 9). Since the soil cores used in this study were intact, many other spatial and temporal sources of variability influenced POM decomposition. For example, while with the use of µCT we were able to quantify the volumes of POM pieces without destroying soil structure, this analysis could not reliably determine types, origins, and decomposition status of the POM fragments used in the analysis.

Our results corroborated the notion that diverse prairie vegetation led to higher soil C than monoculture switchgrass, with concomitantly higher volumes of 30–90 μm radius pores and greater areas of solid-pore interface – the known positive influences on soil C gains. However, that effect was present only in the coarse-textured soils of topographical slopes and coincided with markedly higher belowground root biomass of prairie vegetation. This result suggests that topography can play a substantial role in modulating soil C sequestration benefits of bioenergy cropping systems, likely by modifying root system developments. Surprisingly, new switchgrass growth did not intensify decomposition of POM, but even somewhat decreased it in monoculture switchgrass soil as compared to non-planted controls.

## Supplementary Information

Supplementary Information.

## Abbreviations

POM: Particulate organic carbon
SOC: Soil organic carbon
WFPS: Water filled pore space
X-ray μCT: X-ray computed micro-tomography
