# Supplementary material for: Pore architecture and particulate organic matter in soils under monoculture switchgrass and restored prairie in contrasting topography
Source: Sci Rep. 2021 Nov 9;11:21998. doi: 10.1038/s41598-021-01533-7 (PMC8578609; doi:10.1038/s41598-021-01533-7)
Supplement: Supplementary file 1 — Supplementary Information. [file 41598_2021_1533_MOESM1_ESM.pdf]

Supplementary Information Juyal et al.; submitted to Scientific Reports

Table S1: Results of slicing (aka simple F-tests) of plant system (above) and topography (below) effects on soil texture and particulate organic matter (POM). Shown are *p*-values for system and topography effects, when the level of another factor is fixed. Statistical differences marked in Table 1 are based on the *p*-values presented here.

**Effect**	**Topography**	**Sand (%)**		**Silt (%)**		**Clay (%)**		**POM (%)**	
		F value	p-value	F value	*p*-value	F value	*p*-value	F value	*p*-value
System	Depression	1.04	0.3384	2.92	0.1260	4.79	0.0601	0.63	0.4365
	Slope	10.85	0.0110	12.26	0.0081	0.53	0.4863	0.01	0.9103
**Effect**	**System**	**Sand (%)**		**Silt (%)**		**Clay (%)**		**POM (%)**	
		F value	*p*-value	F value	*p*-value	F value	*p*-value	F value	*p*-value
Topography	Prairie	78.66	<.0001	69.39	<.0001	38.51	0.0003	0.19	0.6681
	Switchgrass	43.48	0.0002	42.72	0.0002	10.81	0.0111	0.10	0.7554

Table S2: Results of one-way ANCOVA analysis for effect of plant system on total C and N across topographical positions with soil sand content used as a covariate. Statistical differences in Figure 2 are based on the *p*-values presented here.

**Type 3 Tests of Fixed Effects**				
**Effect**	**Carbon**		**Nitrogen**	
	**F Value**	*p*-value	**F Value**	*p*-value
**System**	6.64	0.0298	4.73	0.0576
**Sand**	13.58	0.0050	13.65	0.0050

Table S3: Results of slicing (aka simple F-tests) of plant system at given topographies and of topographies at given plant system on soil pore characteristics. Shown are *p*-values for system and topography effects, when the level of another factor is fixed. Statistical differences marked in Table 2 are based on the *p*-values presented here.

**Tests of Effect Slices**									
		**Total porosity (%)**		**Pores >18.2 um (%)**		**Pore connectivity (%)**		**Solid-pore interface (mm^2)**	
**System**	**Topography**	**F Value**	***p*-value**	**F Value**	***p*-value**	**F Value**	***p*-value**	**F Value**	***p*-value**
Prairie		0.01	0.924	0.08	0.785	6.93	0.016	68.04	<0.001
Switchgrass		0.39	0.540	4.21	0.054	0.00	0.98	15.5	0.001
	Depression	1.47	0.240	3.63	0.071	0.71	0.408	0.00	0.971
	Slope	3.83	0.06	0.18	0.67	2.96	0.101	17.28	0.001

Table S4: Results of slicing (aka simple F-tests) of plant system at given topographies and of topographies at given plant system on soil pore size distribution. Shown are *p*-values for system and topography effect, when the level of another factor is fixed. Statistical differences marked in Figure 4 are based on the *p*-values presented here.

**Tests of Effect Slices**											
	**Pore radius**	**30 μm**		**50 μm**		**70 μm**		**100 μm**		**150 μm**	
**System**	**Topography**	**F Value**	***p*-value**	**F Value**	***p*-value**	**F Value**	***p*-value**	**F Value**	***p*-value**	**F Value**	***p*-value**
Prairie		39.08	<.0001	47.17	<.0001	33.89	<.0001	0.28	0.604	5.26	0.033
Switchgrass		4.88	0.039	10	0.005	12.31	0.002	1.24	0.279	6.6	0.018
	Depression	0.14	0.709	0.1	0.750	0.01	0.922	0.58	0.456	0.13	0.722
	Slope	12.51	0.002	10.32	0.004	4.15	0.055	0.8	0.381	0	0.982

Table S5: Summary of soil pore characteristics for the studied prairie and switchgrass systems at the two topographical positions before and after plant growth experiment. Shown are means (n=6) and standard errors for each system and topographical position in control and plant-grown treatments.

**Treatment**	**Topography**	**System**	**Pores >18.2 um (%)**		**Pore connectivity (%)**		**Solid-pore interface (mm^2)**	
		**Treatment**	**Before**	**After**	**Before**	**After**	**Before**	**After**
Plant grown cores	Depression	Prairie	17.5	16.8	93.8	94.0	88.14	87.2
		Switchgrass	17.0	16.1	94.9	94.6	93.4	88.5
	Slope	Prairie	17.8	16.8	88.4	92.0	191.0	170.1
		Switchgrass	17.9	16.6	91.7	93.2	119.6	112.3
		Standard error	0.017		0.01		9.67	
Control cores	Depression	Prairie	17.5	13.6	95.5	88.0	81.6	63.5
		Switchgrass	13.2	10.5	91.4	78.6	78.3	56.9
	Slope	Prairie	17.6	16.9	90.1	91.4	148.9	114.7
		Switchgrass	18.6	16.5	93.7	92.8	178.1	155.1
		Standard error	0.01		0.03		12.71	

There was no significant difference before and after plant growth experiment for any treatment.

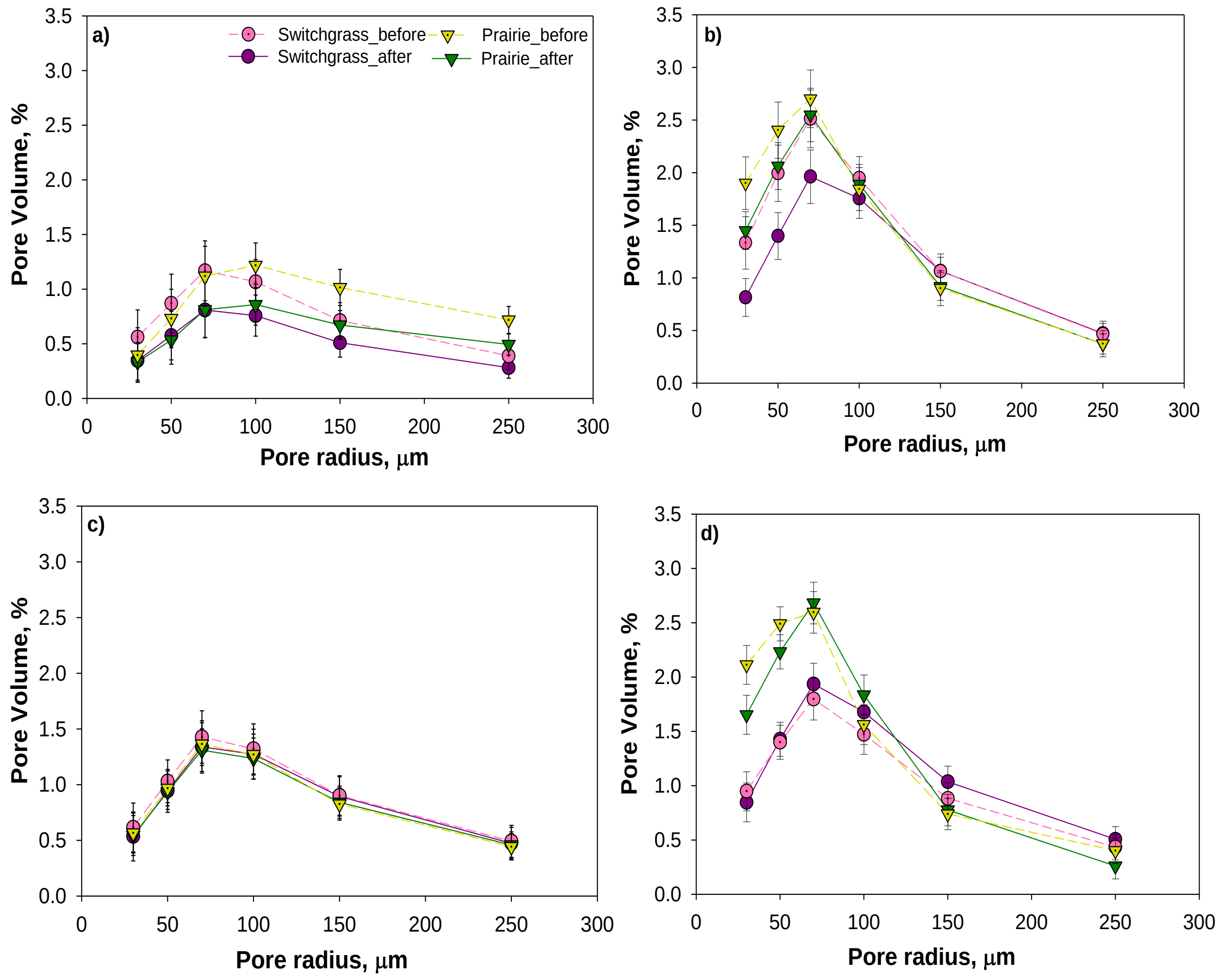

Figure. S1: Pore size distribution for image-based (>18.2 μm) pores for depression (a,c) and slope (b,d) in the before (solid lines) and after (dash lines) plant growth experiment in control(a,b) and plant grown (c,d) cores for prairie (▼) and switchgrass (●) plant systems. Shown are means with error bars representing standard errors (n=3). There was no significant difference before and after plant growth experiment for any treatment.

Table S6: Results of slicing (aka simple F-tests) of plant system and topography at given plant growth treatment and of plant growth treatment at given system and topography on soil pore characteristics. Shown are *p*-values for system, topography and treatment effect, when the level of another factor is fixed. Statistical differences marked in Table 3 are based on the *p*-values presented here.

Tests of Effect Slices								
			Pores >18.2 um (%)		Pore connectivity (%)		Solid-pore interface (mm^2)	
**System**	**Topography**	**Treatment**	**F Value**	***p*-value**	**F Value**	***p*-value**	**F Value**	***p*-value**
Prairie	Depression		3.15	0.0962	1.75	0.2058	2.41	0.1416
Prairie	Slope		0.00	0.9564	0.02	0.9018	0.96	0.343
Switchgrass	Depression		7.5	0.0152	10.96	0.0048	3.42	0.084
Switchgrass	Slope		0.42	0.5273	0.01	0.933	0.25	0.6253
	Depression	Control	4.02	0.0632	5.15	0.0385	0.18	0.674
	Depression	Plant	0.41	0.5298	0.01	0.9234	0.01	0.938
	Slope	Control	0.03	0.8665	0.08	0.7764	7.01	0.0183
	Slope	Plant	0.28	0.6027	0.06	0.8066	9.79	0.0069

Table S7: Results of slicing (aka simple F-tests) of plant system and topography at given plant growth treatment and effect of plant growth treatment at given system and topography on soil pore size distribution. Shown are *p*-values for system, topography and treatment effect, when the level of another factor is fixed. Statistical differences marked in Figure 6 are based on the *p*-values presented here.

			**30 µm**		**50 µm**		**70 µm**		**100 µm**		**150 µm**	
**System**	**Topography**	**Treatment**	**F Value**	***p*-value**	**F Value**	***p*-value**	**F Value**	***p*-value**	**F Value**	***p*-value**	**F Value**	***p*-value**
Prairie	Depression		0.72	0.407	1.69	0.212	2.03	0.174	2.15	0.163	0.92	0.352
Prairie	Slope		1.12	0.306	0.68	0.421	0.45	0.512	0	0.948	0.36	0.557
Switchgrass	Depression		0.45	0.512	1.13	0.305	1.78	0.202	3.08	0.099	3.48	0.081
Switchgrass	Slope		0.07	0.797	0.08	0.786	0.02	0.884	0	0.980	0	0.948
	Depression	Control	0.00	0.955	0.02	0.900	0	0.993	0.14	0.709	0.76	0.397
	Depression	Plant	0.00	0.969	0	0.990	0	0.960	0.01	0.919	0.05	0.822
	Slope	Control	6.25	0.024	4.47	0.051	2.67	0.123	0.27	0.613	0.62	0.441
	Slope	Plant	10.87	0.004	7.1	0.017	4.65	0.047	0.37	0.553	2.12	0.166

Table S8: Results of slicing (aka simple F-tests) of plant system and topography at given plant growth treatment and of plant growth treatment at given system and topography on POM losses. Shown are *p*-values for system, topography and treatment effect, when the level of another factor is fixed. Statistical differences marked in Figure 8 are based on the *p*-values presented here.

Tests of Effect Slices				
**System**	**Topography**	**Treatment**	**F Value**	***p*-value**
Prairie	Depression		0.08	0.7805
Prairie	Slope		0.1	0.7853
Switchgrass	Depression		3.85	0.0685
Switchgrass	Slope		1.23	0.2852
	Depression	Control	0	0.9528
	Depression	Plant	4.84	0.0439
	Slope	Control	0.93	0.3514
	Slope	Plant	0.2	0.6597

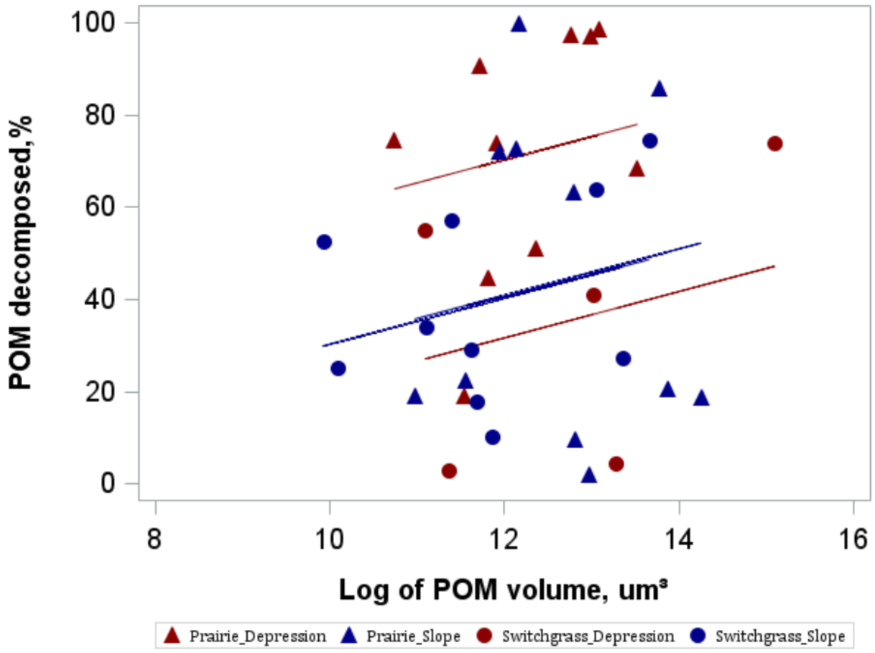

Figure.S2: Relationship between the percent of POM decomposed and size of the POM in plant-grown samples from depressions (red) and slopes (blue) in prairie (▲) and switchgrass (●) system.

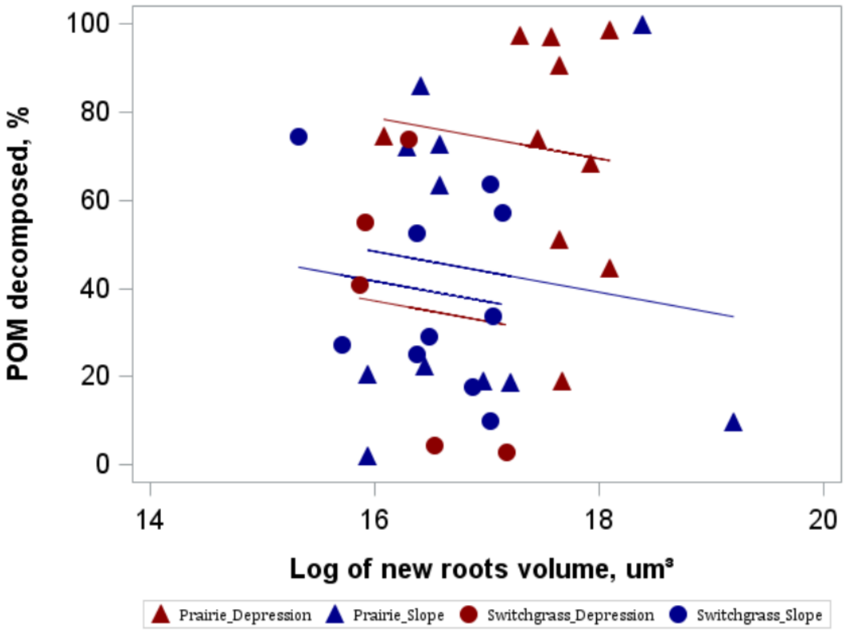

Figure.S3: Relationship between the amount of POM decomposed and the volume of new roots and in plant-grown samples from depressions (red) and slopes (blue) in prairie (▲) and switchgrass (●) system.

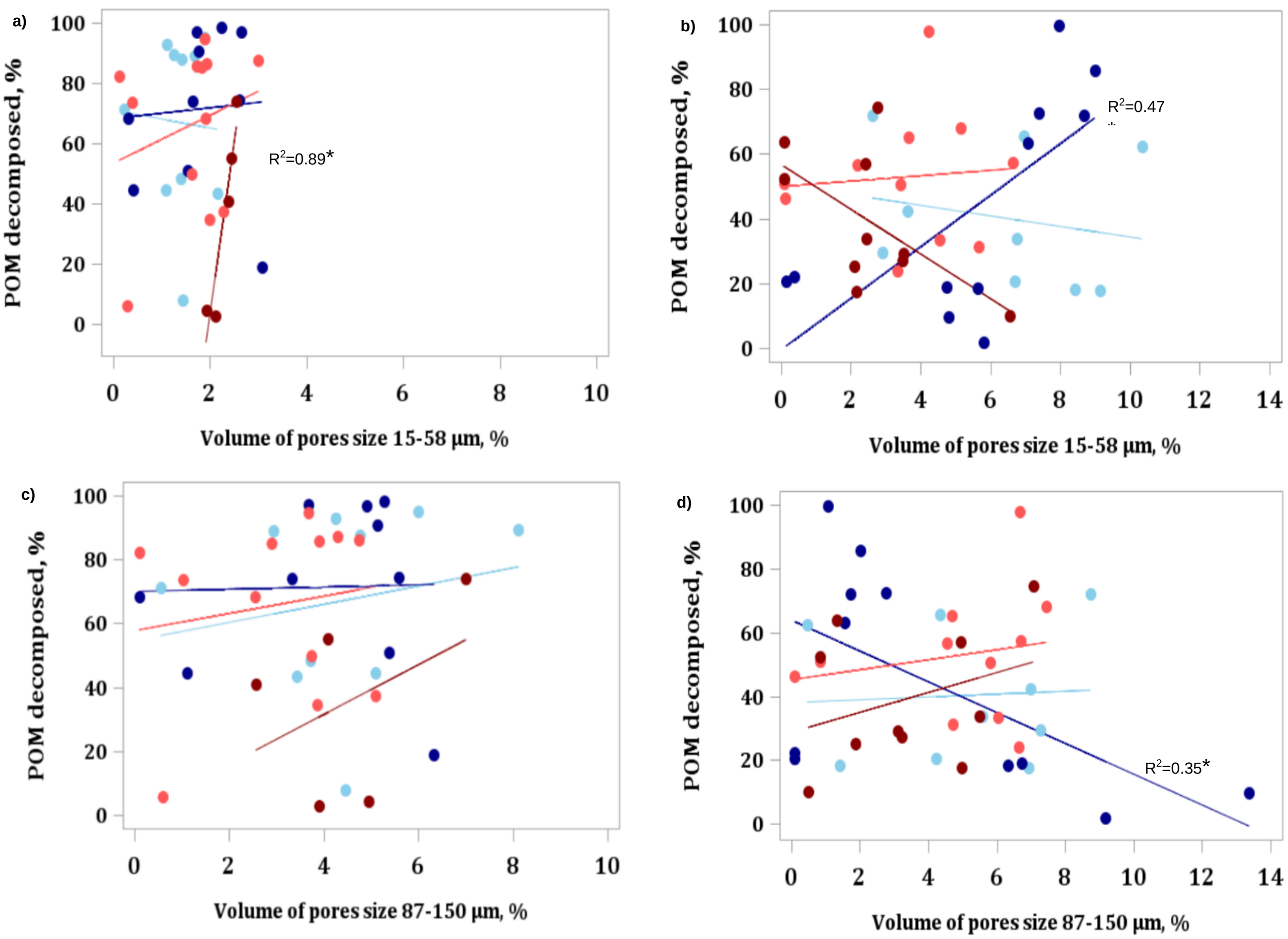

Figure.S4: Relationship between amount of POM decomposed and volume of pores in 15-58 µm and 87-150 µm size range within 5 mm distance from POM fragment in control and plant-grown cores from depressions (a,c) and slopes (b,d) in prairie (blue) and switchgrass (red) system.

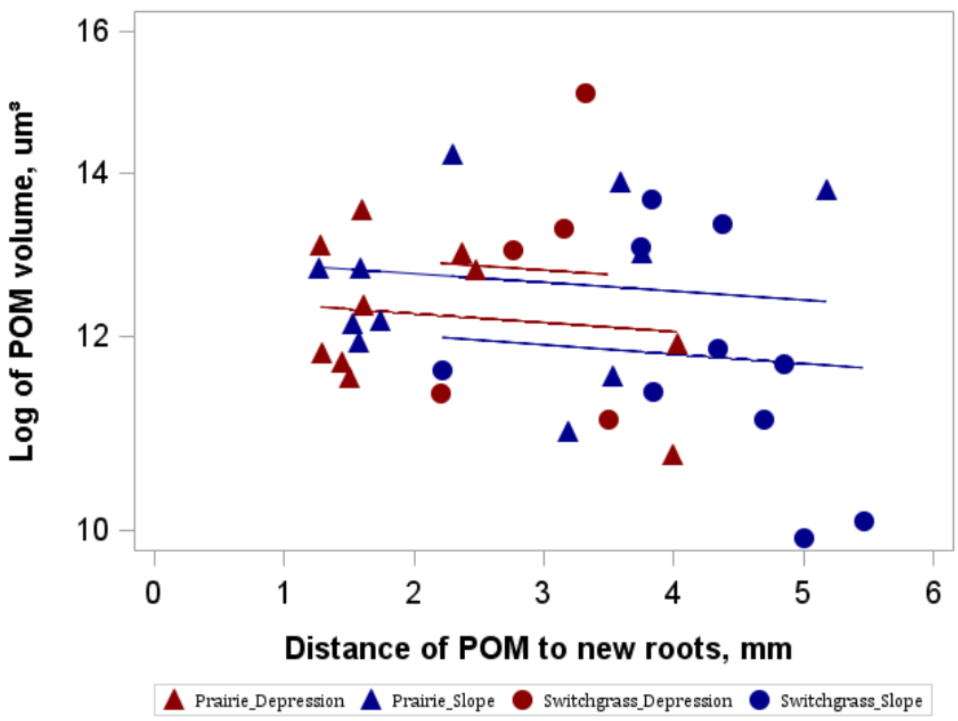

Figure.S5: Correlation between selected POM fragments and distance of POM to new roots in plant-grown samples from depressions (red) and slopes (blue) in prairie (▲) and switchgrass (●) system.
